# Medial prefrontal cortex oxytocin mitigates epilepsy and cognitive impairments induced by traumatic brain injury through reducing neuroinflammation in mice

**DOI:** 10.1038/s41598-023-32351-8

**Published:** 2023-03-30

**Authors:** Wen Chen, Xiaoxiao Man, Yu Zhang, Guangyan Yao, Jing Chen

**Affiliations:** Department of Neurology, Central Hospital Affiliated to Shandong First Medical University, 105 Jiefang Rd, Lixia District, Jinan, 250013 Shandong China

**Keywords:** Brain injuries, Epilepsy

## Abstract

Traumatic brain injury (TBI) is a major risk factor to develop epilepsy and cognitive impairments. Neuropeptide oxytocin has been previously evidenced to produce antiepileptic effects. However, the involvement of central oxytocin in TBI-induced epileptic status and cognitive dysfunctions is not fully elucidated. In this study, we aim to investigate the role of oxytocin on a TBI model followed by seizure induction to clarify whether the epilepsy and cognitive deficits could be mitigated by oxytocin. TBI was established by weight drop and epileptic behaviors were induced by pentylenetetrazole (PTZ) injection in mice. Moreover, oxytocin was microinjected into the medial prefrontal cortex (mPFC) to observe the effects on the epilepsy and cognition. The blood–brain barrier (BBB) function and the neuroinflammation were measured by Evans Blue staining and enzyme-linked immunosorbent assays, respectively. Mice exposed to TBI demonstrate increased vulnerability to PTZ-mediated seizures and cognitive disturbances with a decrease in peripheral and brain oxytocin levels. Additionally, TBI reduces oxytocin, disrupts the BBB permeability and triggers neuroinflammation in mPFC in PTZ-treated mice. Intra-mPFC oxytocin simultaneously mitigates epilepsy and cognitive impairments. Finally, oxytocin restores BBB integrity and reduces mPFC inflammation in PTZ-treated TBI mice. These findings showed that intra-mPFC oxytocin suppressed the seizure vulnerability and cognitive deficits in TBI mice. The normalization of BBB integrity and inhibition of neuroinflammation may be involved in the antiepileptic and cognition-improved effects of oxytocin, suggesting that targeting inflammatory procedure in mPFC may decrease the risk to develop epilepsy and cognitive impairments in individuals previously experienced TBI.

## Introduction

Traumatic brain injury (TBI) is a serious health concern, mainly caused by external force to the head, and is the leading cause of substantial economic burden worldwide. TBI significantly increases the risk of developing epilepsy in the general population^1,2^. Specifically, there are approximately 20% of patients suffering seizures may be due to previous TBI^3^. Beyond this, patients with epilepsy occurred after TBI often present comorbid with cognitive disturbances^4^. An increasing body of evidence unveiled that TBI produces lasting impairments of cognitive processes including episodic memory^5^, attention and executive functioning^6^. However, the unknown neurobiological mechanisms underlying TBI-induced epileptogenesis and cognitive impairment hindered the development of preventions on post-TBI functional and behavioral deficits.

Both animal models and human studies have shown that inflammation induced by TBI may enhance seizure susceptibility^7,8^. For example, prolonged release of inflammatory cytokines interleukin-1β (IL-1β), IL-6 and tumor necrosis factor (TNF) positively correlates with the severity of TBI and with increased-seizure susceptibility and epilepsy^9–12^. Moreover, clinical evidence also showed that there is a causal link between the neuroinflammation and seizure activity according to the findings that pro-inflammatory mediators were altered from brain tissue in patients with epilepsy^13,14^. The role of neuroinflammation in the cortex has also been implicated to be involved in post-TBI epileptogenesis ^15,16^. Neuroinflammation is a major pathological process in the TBI, and it is due to the increased permeability of the blood–brain barrier (BBB) to allow the passage of immune cells that can exacerbate the inflammatory response^17,18^. The BBB is a selectively permeable barrier that is critical in maintaining the brain’s homeostatic environment^19,20^. The association of persistent BBB dysfunction after TBI has been well established^21^. BBB disruption occurs within hours following TBI and may persist for years, and is associated with worse outcomes^22^. However, there is a notable lack of effective intervention to improve the epileptogenesis and cognition, particularly after TBI. Hence, it is important to be able to offer candidate treatments for TBI-induced epilepsy and cognitive decline targeting neuroinflammation and BBB system.

Neuropeptide oxytocin, produced in hypothalamic magnocellular neurons, plays a pivotal role in modulation of key physiological functions^23^. The association between oxytocin and epilepsy has been well clarified in several animal models^24–26^. Importantly, previous investigation has demonstrated that oxytocin maintains BBB integrity and attenuates memory impairment caused by ischemic injury after stroke attack^27^. Improved understanding of alterations of oxytocin function after TBI is critical to identify mechanisms causing these changes and the role of oxytocin signaling in epilepsy and cognitive performance affected by TBI. Evidence suggests oxytocin may exhibit anti-epileptic effects by suppressing glutamatergic neurotransmission in the medial prefrontal cortex (mPFC) layer pyramidal neurons^28,29^. Altered function of oxytocin is predicted to contribute to neuroinflammation and mPFC networks and behavioral consequences of TBI, suggesting that mPFC oxytocin signaling might play a role in the development of epilepsy and cognitive decline induced by TBI.

In the present study, we aim to examine whether mPFC oxytocin affects the vulnerability to pentylenetetrazole (PTZ)-mediated seizures as well as the cognitive performance in mice after TBI. We have determined that mice exposed to TBI demonstrate increased vulnerability to PTZ-mediated seizures and subsequent cognitive disturbances and the normalization of BBB integrity and neuroinflammation may be involved in the antiepileptic and cognition-improved effects of oxytocin.

## Materials and methods

### Animals

Male C57/BL6J mice (8–9 weeks old) were exposed to sham or TBI groups. All mice were housed in groups of four to five in plastic cages and were maintained under standard conditions, with 12-h cycles of light/dark (lights on at 8:00 p.m.) at a temperature of 22 ± 2 °C and humidity (50 ± 5%). Animals had free access to food and water during the entire procedure and were allowed to habituate to testing environment at least one week before the start of experiments. Group assignment, drug administrations, surgery procedure and behavioral tests were performed between 10:00 a.m. and 5:00 p.m. during the active phase of animals. The blind experimenters who had no information of the experimental conditions conducted the data collection and data processing. All animal experiments were performed in accordance with the National Research Council's Guide for the Care and Use of Laboratory Animals. The protocols of animal experiments were approved by the Institutional Animal Care and Use Committee of Shandong First Medical University (approval number: GZR2020-001-01). The anaesthesia (inhalation of isoflurane) and euthanasia (inhalation of carbon dioxide) of animals were carried out in accordance with the American Veterinary Medical Association (AVMA) Guidelines for the Euthanasia of Animals (2020). The study is reported in the manuscript follows the recommendations in the ARRIVE guidelines.

### Traumatic brain injury (TBI) procedure

The TBI model was established using free weight drop model in mice according to previous studies with minor modifications^30–32^. Briefly, mice were anesthetized by 2–5% isoflurane inhalation before they were placed on a stereoscopic device with an 80-cm high plastic guide tube (13 mm in diameter) vertically over the head of the mouse^33,34^. TBI was induced by a 50 g metal weight dropped down through the guide tube onto the center of the head. Sham mice used for control received the same anesthesia as TBI mice but not subjected to head impact by the falling weight.

### Pentylenetetrazole (PTZ) induced seizure model

Seven days after TBI, mice received intraperitoneal injection of saline or PTZ at a sub-convulsive dose of 50 mg/kg (i.p.) to determine seizure susceptibility induced by TBI^35^. PTZ (1,5-pentamethylenetetrazole, purity ≥ 99%; Sigma-Aldrich, St. Louis, USA) was dissolved in sterile 0.9% saline (2.5 mg/ml) and was prepared freshly on the injection day. PTZ is a gamma-amino butyric acid (GABA)-A receptor antagonist and is widely used for pharmacological induction in rodents to investigate the development and prevention of epilepsy^36^. Each mouse received a single administration of PTZ at the volume of 0.2 ml/10 g weight and was immediately transferred to a transparent cage. A modified Racine Scale for the behavioral scoring of severity of PTZ-induced seizures in mice was utilized^37^. In details, the seizure severity was defined within 30 min after PTZ injection by the score as 0: normal behavior; 1: whisker trembling; 2: sudden behavior arrest; 3: facial jerking; 4: neck jerks; 5: clonic seizure; 6: clonic, tonic–clonic seizure; 7: clonic, tonic–clonic seizure and wild jumping; 8: tonic extension, possibly leading to respiratory arrest and death.

### T-maze test

We used spontaneous alternation T-maze test to evaluate cognitive function in TBI mice as performed previously^38,39^. A T-maze was made of black plastic and consisted of a central arm (50 cm × 10 cm, walls 15 cm height) and two lateral arms (32 cm × 10 cm, walls 15 cm height). During the training process, mice were placed in the start area and allowed to acclimatize for 5 min with all doors open. The testing process was performed 24 h later. Briefly, mice were allowed to ambulate up the starting arm and make a choice of the left or right of the lateral arms. Once entering, the door to the arm was closed and the mouse was confined in the chosen arm for 30 s. Mice were then removed and returned to the central arm for the next trial. If the mouse did not make a choice within 300 s, the trial is ended. There are in total eight successive free trials in the T-maze for each mouse. The chosen arm was recorded and the percentage of spontaneous alternations was calculated by number to the novel arm visits over the eight trials.

### Novel object recognition test

We used the novel object recognition test (NORT) to assess the nature of mice by exploring novel or unfamiliar objects and differentiate them from familiar objects^40^. Specifically, one day before the training session, mice were allowed to explore an open-field arena (42 cm × 42 cm × 42 cm) without objects for 5 min. The next day, two identical objects were placed in opposite and symmetrical corners of the arena. The mice were then placed in the center of the arena with their backs to the objects for 5 min (training session) and thereafter returned to their home cages. After 1 h, one of the familiar objects was replaced with a different one (novel object); the mice were returned into the arena again and were allowed to explore the objects for 5 min (testing session), during which the time that the mice spent with both objects was recorded. Data were presented as: recognition ratio = time (s) spent with the novel object/(time spent with the novel object + time spent with the familiar object)^41,42^.

### Social recognition test

The social recognition test was performed as described previously with a minor modification^43,44^. The group-housed test mice were individually housed for one hour before the beginning of test. During the habituation session, mice were allowed to freely explore the apparatus composed of a three-chamber apparatus composed by an inner chamber (15 cm × 40 cm × 24 cm) and two outer chambers (20 cm × 40 cm × 24 cm) for 5 min. For the social recognition test, an unfamiliar male CD1 mouse (unfamiliar mouse 1), which did not have access to the test mouse, was randomly placed in one of two chambers enclosed into a small empty Plexiglas cage (7 cm × 10 cm × 24 cm) that enabled visual, auditory and olfactory contact but without direct physical touching and fighting. The test mouse was introduced to the apparatus and allowed to explore all three chambers for 10 min. After a 5-min inter-session break at the end of 10-min exploration, the test mouse was returned to the chambers. Subsequently, a second unfamiliar male CD1 mouse (unfamiliar mouse 2) was placed in the previously empty chamber so that the test mouse had the freedom to explore either the unfamiliar mouse 1 or the novel mouse 2. The test animals were observed in a 10 min social recognition test. The time spent and the number of entries into the different chambers by the test mouse in each session was recorded. The ratio of mouse 2 to mouse 1 in the time spent and the number of entries respectively was used to determine social recognition memory. We also assessed the distance travelled during the 10-min session to exclude the possible effects of locomotion on the exploratory activity of the test mouse. The chambers were cleaned with 30% ethanol between sessions to eliminate the odour cues of previous mouse.

### Blood–brain barrier integrity measurement by EB quantification

To determine the BBB integrity, Evans Blue (EB) extravasation method was used as previously reported^45,46^. Briefly, animals were injected intravenously in the tail with 2% EB solution (CAS 314-13-6, Sigma-Aldrich) at the dose of 0.2 ml/10 g bodyweight. After 2 h venous injection, mice were transcardially perfused with 150 ml saline for 15 min. The brain was removed from the skull and one of the hemispheres was homogenized in phosphate buffer solution, and diluted with trichloroacetic acid and centrifuged for 30 min at 3500 rpm. The supernatant was collected and diluted with ethanol. Samples were measured in triplicate at 610 nm on a plate reader spectrophotometer (Thermo Scientific Multiskan SkyHigh). The results were reported in ratio of naïve mice for the absorbance of EB.

### Tissue sample preparation and Western blot

The tissues of mouse mPFC were collected and homogenized (5000 rpm, 2 × 30 s, 10 s break) with a high-speed tissue homogenizer after being lysed with RIPA lysis buffer, in which protease inhibitor cocktail was added to inhibit protein degradation during the process. Afterward, the homogenate was centrifuged at 10,000*g* at 4 °C for 10 min. Supernatant were transferred to a new tube and the protein concentrations of all samples were determined using the BCA assay kit (Beyotime Biotechnology, Shanghai, China). The samples were diluted with RIPA lysis buffer to normalize the protein concentration. All of the above procedures were performed on ice to keep the low temperature at 0–4 °C. Subsequently, samples were mixed with 4 × loading sample buffer and boiled at 95 °C for 5 min. All these tissue lysates were stored at − 80 °C before use. Equal amounts of protein (20 μg) were loaded into the wells of 12% Protein Gels (Meilun Biotechnology, Dalian, China) for 45–60 min at 120 V. Proteins were electrophoretically transferred to Immobilon-P transfer membranes (Millipore, Bedford, MA, USA) at 0.25 A for 2 h. After transfer, the membranes were blocked with 5% BSA in TBST (Tris-buffered saline plus 0.05% Tween-20, pH 7.4) for 1 h at room temperature. Following blocking, membranes were incubated with the primary rabbit anti-Claudin-5 monoclonal antibody (1:1000, ab131259, Abcam) or mouse β-actin monoclonal antibody (1:3000, TA-09, Zhongshan Jinqiao Biotechnology, Beijing, China) in TBST plus 5% milk overnight at 4 °C with a gentle shaking system. The next day, blots were washed in TBST buffer four times with 6 min of each time, and were incubated for 1 h at room temperature on a shaker with horseradish peroxidase (HRP)-conjugated goat anti-mouse IgG (1:3000, ZB-2305) or goat anti-rabbit 1:2000, ZB-2301, Zhongshan Jinqiao Biotechnology, Beijing, China) IgG secondary antibody. Blots were washed four times for 6 min with TBST and were incubated with a layer of Super Signal enhanced chemiluminescence substrate mixture (Pierce Biotechnology, Rockford, IL, USA) for 1 min at room temperature. The blots were directly imaged using ChemiDoc™ MP (Bio-Rad, CA, USA) and the band intensities for Claudin-5 were normalized to β-actin protein expressions with Image Lab Software V5.1 (Bio-Rad, CA, USA).

### Measurement of S100β, inflammatory cytokines, and oxytocin levels using enzyme-linked immunosorbent assays (ELISA)

ELISA for mouse S100β were carried out with serum according to manufacturer’s instructions (R&D). Briefly, 100 μl of serum were applied on 96-well plates pre-coated with monoclonal anti-S100β overnight at room temperature. After washing for three times, 100 μl of biotin labeled antibody was added and incubation continued for 1 h. The plate was washed and 100 μl of streptavidin-HRP conjugate was added and the plate was incubated for a further 30 min in the dark. The addition of 100 μl of the substrate and stop solution represented the last steps before the reading of absorbance (measured at 450 nm) on a microplate reader. S100β levels in the samples were calculated using a standard curve of S100β and expressed as pg/ml.

To measure the levels of inflammatory cytokines in mice, medial prefrontal cortex homogenates were collected in PBS by centrifugation for 5 min at 5000*g* at 4 °C, and the supernatant was collected. The levels of TNF-α, IL-6, and IL-1β were analyzed using commercially available ELISA kits, according to the manufacturer’s instructions. The ELISA Kit of mouse TNF-α (MM-0132M2), IL-6 (MM-0163M2), and IL-1β (MM-0040M2) were purchased from Jiangsu Meimian Biological Technology Co. Ltd (Jiangsu, China). All tests were performed in triplicates and expressed as pg/ml.

To measure the plasma oxytocin levels, blood was collected from each mouse and centrifuged at 4000 rpm for 15 min under 4 °C. Oxytocin levels were determined using ELISA kit (Enzo Life, USA) following the manufacturer’s instructions. For the measurement of oxytocin levels in the mPFC, mouse brains were quickly extracted and the tissue punches of mPFC were diluted with the assay buffer in the ELISA kit and homogenized using a high-throughput tissue homogenizer. The tissue homogenate was centrifuged for 15 min at 4 °C and 13,000*g*, and the supernatant was collected. Brain tissue extraction oxytocin concentrations were measured using the oxytocin ELISA kit, following the manufacturer's instructions. The total oxytocin content of each sample was expressed as pg/ml.

### Stereotactic surgery and intra-mPFC microinjection

Mice were anesthetized by 2–5% isoflurane inhalation before guide cannulae (OD 0.41 mm × ID 0.25 mm) were implanted into their brains using the following stereotaxic coordinates for mPFC: anterior/posterior, + 1.75 mm; medial/lateral, ± 0.75 mm; dorsal/ventral, − 2.65 mm at a 15° angle^47^. After surgery, the general health conditions of all mice were monitored and they were allowed a 7-day recovery before subsequent treatment. Mice were intracranially microinjected with either oxytocin (Biorbyt, orb71832, 5 μg/μl) or its vehicle (saline) using 10 μl Hamilton syringes (Hamilton, Reno, NV) that were connected via polyethylene-50 tubing (OD 0.61 × ID 0.28) to injectors (OD 0.21 × ID 0.11, RWD Lifescience, Shenzhen, China) extending 0.8 mm beyond the tip of the cannula. A total volume of 0.1 μl oxytocin was infused into the each side of mPFC over 5 min, and the injection syringe was left in place for an additional 5 min to allow for entire diffusion. For intra-mPFC infusion, oxytocin or its vehicle (saline) was infused at a dose of 1 μg/side/mouse. In all groups, total infusion volume was 0.2 μl per mouse. The dose of oxytocin for microinjection into the mPFC is referred to previous study in a mouse model of fear memory, in which dose-window was confirmed when delivered directly into the amygdala^48^. Data from mice with incorrect placements assessed using Nissl staining of the bilateral injection cannula were excluded from the statistical analysis.

### Statistical analysis

Data are presented as the means ± SEM. Statistical analysis was performed using the Prism 8 software (GraphPad 8, San Diego, USA). The homogeneity test of variance was performed before data analysis. For multiple comparisons, one-way or two-way analysis of variance (ANOVA) were used with the appropriate between- and within-subjects factors for the different experiments with a 2 × 2 analysis of variance was performed to assess the interaction between two factors (TBI × PTZ or PTZ × oxytocin with details in “Results”). Tukey’s test was used for post hoc comparisons. Non-parametric analysis followed by Kruskal–Wallis test was used for Racine Scales data. A Pearson correlation analysis was performed on BBB integrity and seizures activity and cognitive function. Differences were considered significant for values of *P* < 0.05. Researchers were blinded to the group assignment of animals. Sample sizes were identified according to previous studies in this field. Statistical parameters, including n, and *P* values, and the analytical method used for each experiment, are described in the figure legends.

### Ethics statement

All of procedures performed in this study involving animals were in accordance with institutional animal care committee for animal surgery and ethical international guidelines for the care and use of laboratory animals. Research procedure the Institutional Animal Care and Use Committee of Shandong First Medical University.

## Results

### TBI increases the vulnerability to PTZ-mediated seizures

**Figure 1:**
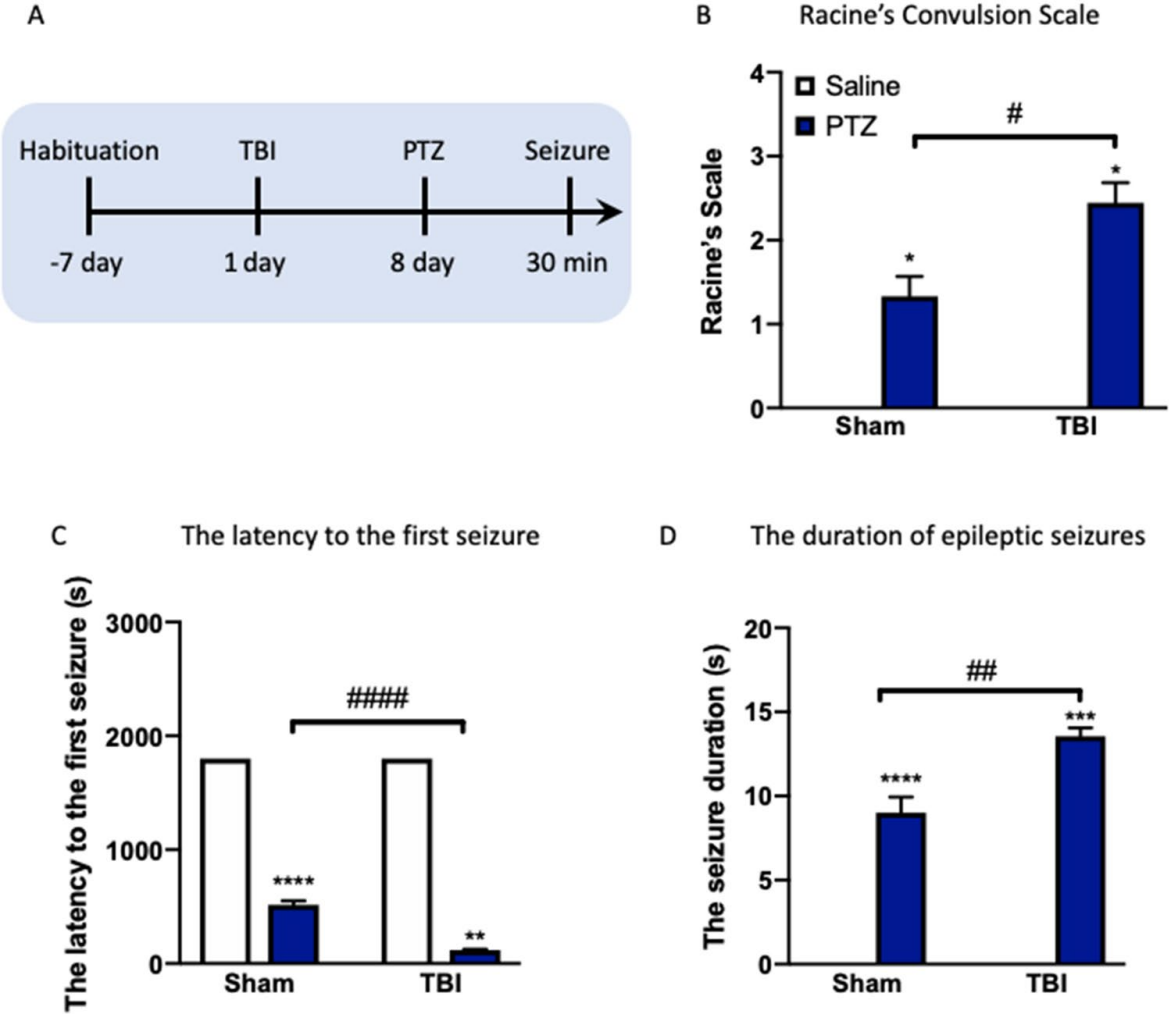
TBI increases the vulnerability to PTZ-mediated seizures in mice. (**A**) Experimental timeline for TBI establishment, PTZ treatment and seizure test. (**B**) The Racine’s Scale. (**C**) The latency to the first seizure. (**D**) The duration of epileptic seizures. Data are presented as mean ± SEM. *P < 0.05, **P < 0.01, ***P < 0.001, ****P < 0.0001, compared with saline group; ^#^*P* < 0.05, ^##^*P* < 0.01, ^####^*P* < 0.0001, compared with corresponding sham group. Two-way ANOVA followed by Turkey’s test. Non-parametric analysis followed by Kruskal–Wallis test was used for Racine’s Scales data. n = 8–9 mice for each group. *TBI* traumatic brain injury, *PTZ* pentylenetetrazole.

Following 7-day recovery from TBI in C57BL/6J mice, we first injected mice with PTZ (50 mg/kg, i.p.) or saline and measured the Racine’s Scale, the latency to the first seizure, and the duration of epileptic seizures to assess a seizure phenotype (Fig. 1A). PTZ injection led to a significant elevation of Racine’s Scale in mice received weight drop relative to saline group (*P* < 0.05, Fig. 1B), and induced a significant seizure in TBI mice relative to sham mice without exposure to TBI (*P* < 0.05, Fig. 1B). Two-way ANOVA analysis showed significant effects of TBI (*F*_(1, 31)_ = 124.6, *P* < 0.0001) and PTZ (*F*_(1, 31)_ = 6911, *P* < 0.0001) and a significant effect of TBI × PTZ interaction (*F*_(1, 31)_ = 124.6, *P* < 0.0001) on the latency to the first seizure. Further behavioral measurements revealed TBI decreased the latency to the first seizure (*P* < 0.0001, Fig. 1C), a marker showing increased seizure activity. A reduction in the latency to the first seizure was observed in PTZ-injected TBI mice compared with saline mice (*P* < 0.05, Fig. 1C). For the duration of epileptic seizures, two-way ANOVA revealed significant effects of TBI (*F*_(1, 31)_ = 17.07, *P* < 0.001) and PTZ (*F*_(1, 31)_ = 418.5, *P* < 0.0001) and a significant effect of TBI × PTZ interaction (*F*_(1, 31)_ = 17.07, *P* < 0.001). Post-hoc analysis showed that elevated duration of epileptic seizures was observed in PTZ-treated TBI mice compared with saline mice (*P* < 0.05, Fig. 1D). These results indicate that previous TBI exposure increases the vulnerability to PTZ-mediated seizures in a mouse model of experimental epilepsy.

### TBI induces cognitive impairments in PTZ-treated mice

**Figure 2:**
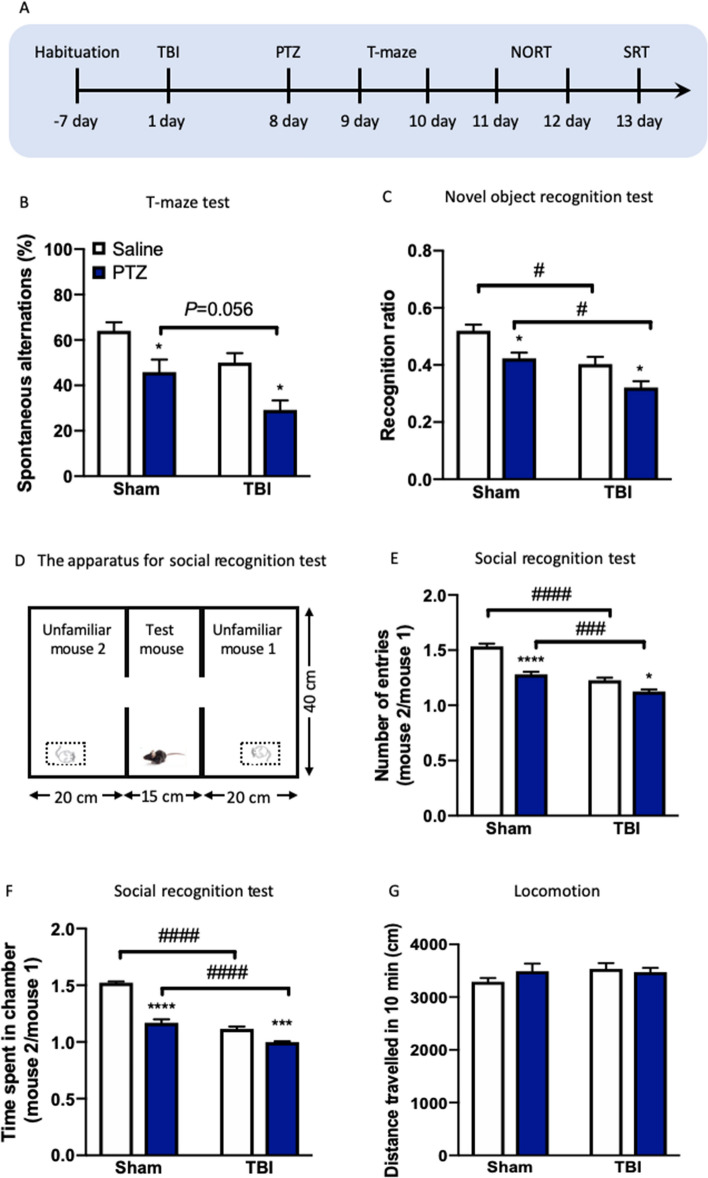
TBI induces cognitive impairments in PTZ-treated mice. (**A**) Experimental timeline for T-maze, NORT and SRT. (**B**) The spontaneous alterations in T-maze test. (**C**) The recognition ratio in NORT. (**D**) Schematic of SRT paradigm. (**E**) Time spent in chamber in SRT. (**F**) The number of entries in SRT. (**G**) Distance travelled in SRT. Data are presented as mean ± SEM. *P < 0.05, ***P < 0.001, ****P < 0.0001, compared with sham group; ^#^*P* < 0.05, ^###^*P* < 0.001, ^####^*P* < 0.0001, compared with saline group. Two-way ANOVA followed by Turkey’s post hoc test. n = 8–9 per group. *TBI* traumatic brain injury, *PTZ* pentylenetetrazole, *NORT* novel object recognition test, *SRT* social recognition test.

We next examine the effects of exposure to TBI on the cognitive performance using T-maze, novel object recognition test (NORT) and social recognition test (SRT) after a mild TBI in mice (Fig. 2A). Two-way ANOVA revealed significant effects of TBI (*F*_(1, 31)_ = 11.72, *P* < 0.01) and PTZ (*F*_(1, 31)_ = 18.94, *P* < 0.001) on the spontaneous alterations in T-maze test. The behavioral results showed that mice received weight drop exhibited a trend for reduction in spontaneous alterations in T-maze test compared to sham mice (*P* < 0.05, Fig. 2B), which indicates a decline of cognitive performance. PTZ-treated mice showed a decrease trend in spontaneous alterations in TBI rather than sham (*P* = 0.056, Fig. 2B). Two-way ANOVA revealed significant effects of TBI (*F*_(1, 31)_ = 24.68, *P* < 0.0001) and PTZ (*F*_(1, 31)_ = 16.49, *P* < 0.001) on the recognition ratio in the NORT. TBI mice also induced a reduction in the recognition ratio (*P* < 0.05, Fig. 2C), suggesting an impaired cognition relative to sham mice. While PTZ addition resulted in a worsen cognitive performance in TBI mice according to the recognition ratio of the NORT when compared to saline-treated sham mice (*P* < 0.05, Fig. 2C). Two-way ANOVA revealed significant effects of TBI (*F*_(1, 31)_ = 218.4, *P* < 0.0001) and PTZ (*F*_(1, 31)_ = 145.0, *P* < 0.0001) and a significant effect of TBI × PTZ interaction (*F*_(1, 31)_ = 36.91, *P* < 0.0001) on time spent in chamber in the SRT (Fig. 2D). Two-way ANOVA also revealed significant effects of TBI (*F*_(1, 31)_ = 102.0, *P* < 0.0001) and PTZ (*F*_(1, 31)_ = 59.97, *P* < 0.0001) and a significant effect of TBI × PTZ interaction (*F*_(1, 31)_ = 11.20, *P* < 0.01) on numbers of entries in the SRT. Moreover, in the SRT, mice received TBI also showed an increase in time spent in chamber (*P* < 0.001, Fig. 2E) and numbers of entries (*P* < 0.05, Fig. 2F) compared to sham mice. Expectedly, PTZ administration aggravated cognition reflected by the further decrease in time spent in chamber (*P* < 0.0001, Fig. 2E) and numbers of entries (*P* < 0.001, Fig. 2F) in the SRT. We further found that PTZ did not change the locomotion as assessed by distance travelled in social recognition test in both sham (*P* < 0.0001, Fig. 2G) and TBI mice (*P* < 0.001, Fig. 2G). Similar to what we found in epileptic measurements, previous TBI exposure seriously impacts the cognitive function in mice exposed to PTZ treatment.

### TBI mice treated with PTZ show reduced oxytocin levels at the periphery and in the mPFC

**Figure 3:**
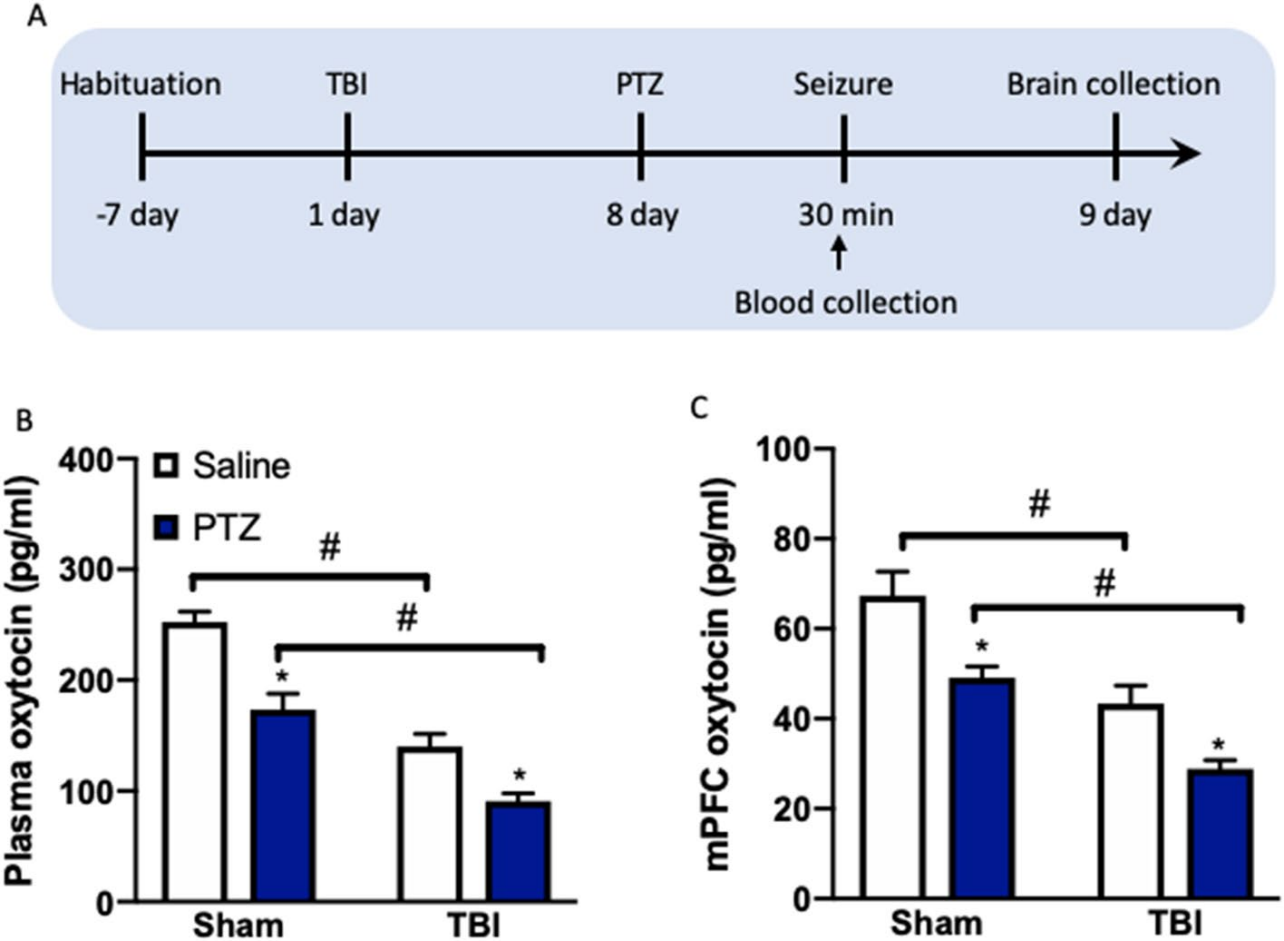
TBI mice treated with PTZ show reduced oxytocin levels at the periphery and in the mPFC. (**A**) Experimental timeline for treatment and assignment. (**B**) The plasma oxytocin levels. (**C**) The mPFC oxytocin levels detected by ELISA. Data are presented as mean ± SEM. *P < 0.05, compared with saline group; ^*#*^*P* < 0.05, compared with corresponding sham group. Two-way ANOVA followed by Turkey’s test. n = 8 mice for each group. *TBI* traumatic brain injury, *PTZ* pentylenetetrazole.

To determine the possible link of the neuropeptide oxytocin and seizures, we performed a direct assessment for the endogenous levels of oxytocin in PTZ-treated TBI mice (Fig. 3A) and found that both the peripheral and mPFC oxytocin concentrations were decreased. Analysis of the effect of TBI and PTZ on plasma oxytocin by two-way ANOVA revealed a significant main effect of TBI (*F*_(1,28)_ = 78.96, *P* < 0.0001) and PTZ (*F*_(1,28)_ = 34.19, *P* < 0.0001). Multiple comparisons by Tukey post-hoc test to the saline-treated sham and TBI revealed that PTZ reduced plasma oxytocin levels (*P* < 0.05) with a much lower oxytocin in TBI mice within PTZ administration groups (*P* < 0.05, Fig. 3B). Consistently, a subsequent analysis of the effect of TBI and PZT on mPFC oxytocin by two-way ANOVA revealed a significant main effect of TBI (*F*_(1,28)_ = 35.57, *P* < 0.0001) and PTZ (*F*_(1,28)_ = 19.39, *P* < 0.001). Tukey post-hoc test to the saline treated sham and TBI revealed that PTZ reduced mPFC oxytocin levels (*P* < 0.05) with a much lower oxytocin in TBI mice within PTZ administration groups (*P* < 0.05, Fig. 3C).

### TBI disrupts the BBB permeability in PTZ-treated mice

**Figure 4:**
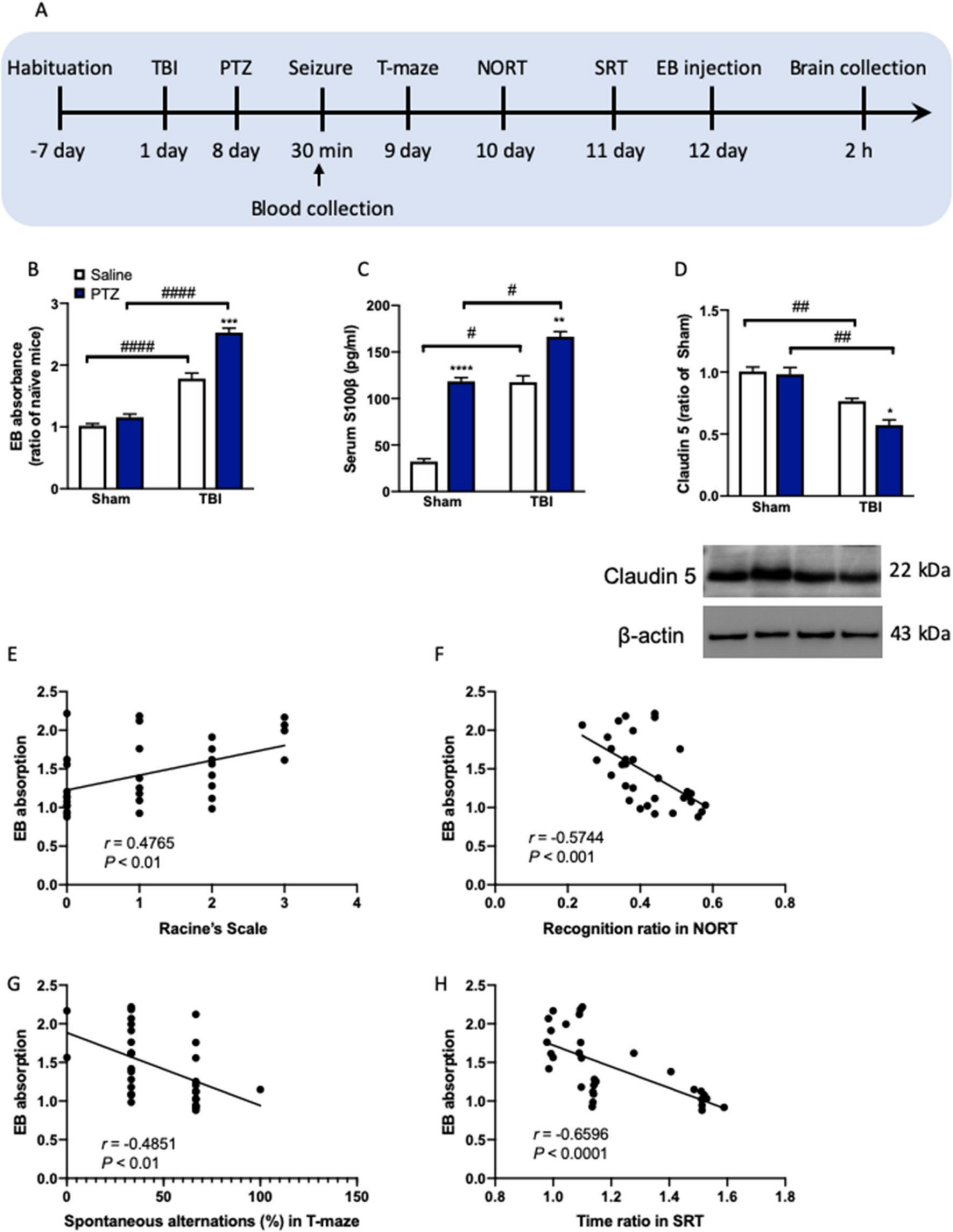
TBI disrupts the BBB permeability in PTZ-treated mice. (**A**) Experimental timeline for treatment and assignment. (**B**) The EB absorbance. (**C**) The serum S100β levels measured by ELISA. (**D**) The Claudin-5 levels with representative images in Western blot on the bottom. Data are presented as mean ± SEM. *P < 0.05, **P < 0.01, ***P < 0.001, ****P < 0.0001, compared with saline group; ^#^*P* < 0.05, ^##^*P* < 0.01, ^####^*P* < 0.0001, compared with corresponding sham group. Two-way ANOVA followed by Turkey’s test. n = 8 mice for each group. Relationship between BBB integrity and seizure activity in PTZ-treated TBI by Pearson correlation analysis shown by (**E**) A positive negative relationship between EB absorbance and Racine’s Scale in seizure (*r* = 0.4765, *P* < 0.01). Relationship between BBB integrity and cognitive performance in PTZ-treated TBI by Pearson correlation analysis shown by (**F**) A negative relationship between EB absorbance and recognition ratio in NORT (*r* = − 0.5744, *P* < 0.001), (**G**) A negative relationship between EB absorbance and spontaneous alterations in T-maze test (*r* = − 0.4581, *P* < 0.01), and (**H**) A negative relationship between EB absorbance and time ratio in SRT (*r* = − 0.6596, *P* < 0.0001). n = 32 mice for this group. *TBI* traumatic brain injury, *PTZ* pentylenetetrazole, *NORT* novel object recognition test, *SRT* social recognition test.

Previous evidence showed a direct correlation between human epilepsy and BBB permeability^49^. To determine whether seizure activity directly drives changes to BBB integrity, we evoked seizures in TBI mice with PTZ and subject mice to the following seizure activity, T-maze, NORT and SRT (Fig. 4A). We first investigated brain-blood vascular leakage by EB staining and assessment of the levels of serum S100β which is a marker of BBB disruption and has previously been reported to be elevated in epilepsy^50^. Two-way ANOVA revealed significant effects of TBI (*F*_(1, 28)_ = 262.0, *P* < 0.0001) and PTZ (*F*_(1, 28)_ = 44.52, *P* < 0.0001) and a significant effect of TBI × PTZ interaction (*F*_(1, 28)_ = 21.14, *P* < 0.0001) on the EB absorbance. EB staining showed TBI increased BBB leakage in both saline and PTZ group compared to sham mice (*P* < 0.0001, Fig. 4B). TBI mice exposed to PTZ demonstrated a higher level of EB staining when compared to saline-treated mice (*P* < 0.001, Fig. 4B). Two-way ANOVA revealed significant effects of TBI (*F*_(1, 28)_ = 176.8, *P* < 0.0001) and PTZ (*F*_(1, 28)_ = 180.6, *P* < 0.0001) and a significant effect of TBI × PTZ interaction (*F*_(1, 28)_ = 13.76, *P* < 0.001) on the serum S100β levels. Serum analysis of S100β showed elevated levels of PTZ injection within 30 min when seizures were evoked (*P* < 0.01, Fig. 4C). Western blot analysis of mPFC protein samples from the brain of TBI mice revealed levels of Claudin-5 protein were significantly decreased in both saline and PTZ mice from sham group (*P* < 0.01, Fig. 4D). Two-way ANOVA revealed significant effects of TBI (*F*_(1, 28)_ = 63.37, *P* < 0.0001) and PTZ (*F*_(1, 28)_ = 7.067, *P* < 0.05) and a significant effect of TBI × PTZ interaction (*F*_(1, 28)_ = 4.412, *P* < 0.05) on the Claudin-5 protein. The full-length uncropped Western blots are shown in Supplementary Information. Moreover, a Pearson correlation analysis was performed on BBB integrity and seizures and cognitive function to clarify the potential link. EB absorption positively correlated with Racine’s Scale in seizure with a correlation coefficient of *r* = 0.4765 shows a positive relationship between these two values (*P* < 0.01, Fig. 4E), suggesting that the higher BBB permeability and increased seizure activity were noted. We further observed that EB absorption was negatively correlated with recognition ratio in NORT (*r* = − 0.4765, *P* < 0.001, Fig. 4F), spontaneous alternations in T-maze (*r* = − 0.48515, *P* < 0.01, Fig. 4G), and time ratio in SRT (*r* = − 0.6596, *P* < 0.0001, Fig. 4H), showing a significant relationship between BBB integrity and cognitive performance.

### TBI triggers neuroinflammation in mPFC in PTZ-treated mice

**Figure 5:**
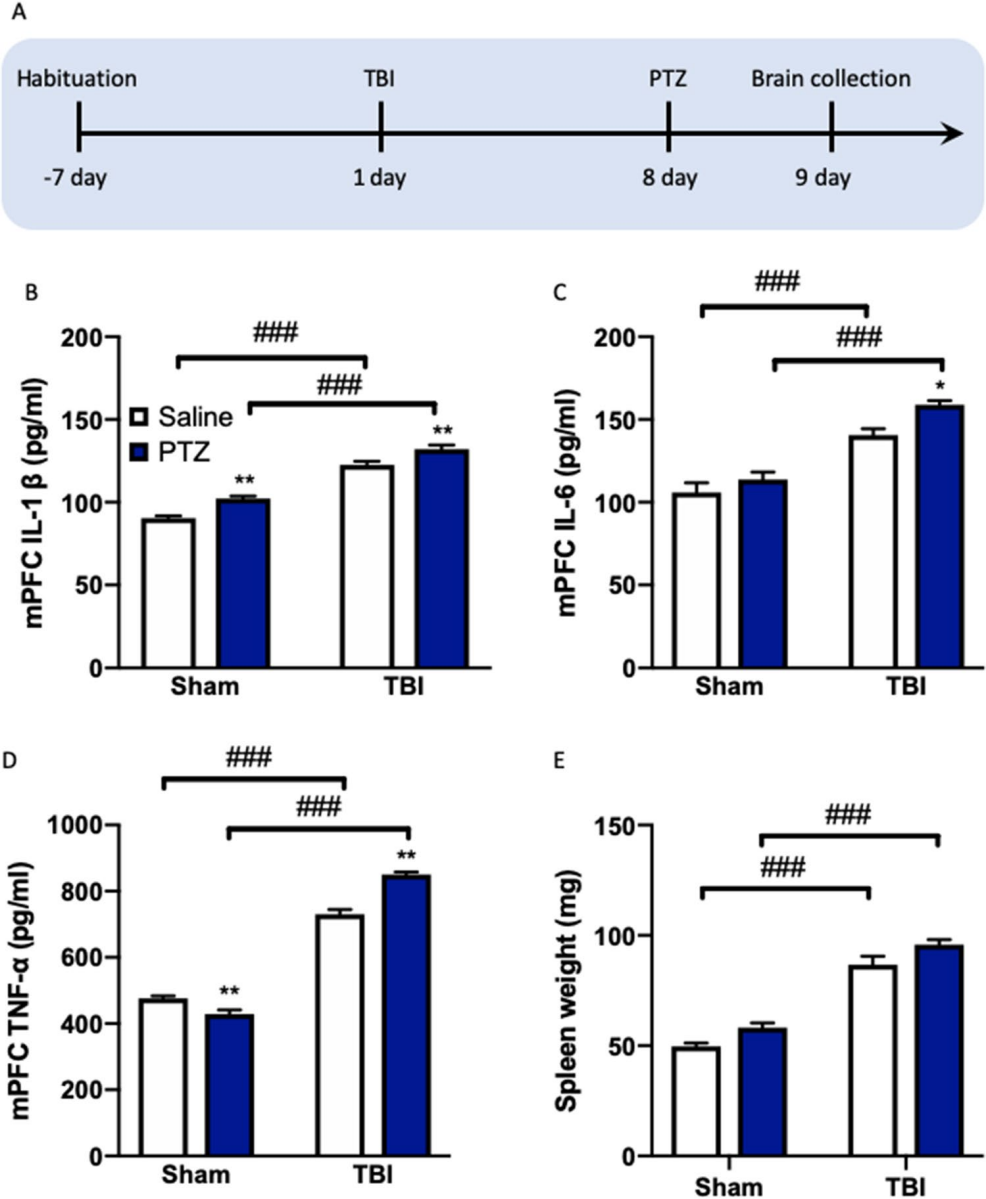
TBI triggers neuroinflammation in the mPFC in PTZ-treated mice. (**A**) Experimental timeline for treatment and assignment. The levels of (**B**) IL-1β, (**C**) IL-6 and (**D**) TNF-α in the mPFC measured by ELISA Kit. (**E**) The spleen weight of each mouse. Data are presented as mean ± SEM. *P < 0.05, **P < 0.01, compared with saline group; ^###^*P* < 0.001, compared with corresponding sham group. Two-way ANOVA followed by Turkey’s post hoc test. n = 8 mice for each group. *TBI* traumatic brain injury, *PTZ* pentylenetetrazole.

The above results showed that impaired BBB stability is associated to the PTZ-evoked epileptic behavior and cognitive performance of TBI mice. A recent study showed that restoration of Claudin-5 levels attenuates seizures and neuroinflammation^49^, which suggest that neuroinflammation altered by BBB dysfunction may participate in TBI triggered seizure activity and cognitive decline. Therefore, we tested whether the levels of inflammatory cytokines in the mPFC were affected by BBB leakage in PTZ-treated mice (Fig. 5A). Exposure to PTZ treatment after TBI induced increases in IL-1β, IL-6 and TNF-α in the mPFC (Fig. 5B–D). Data analyses from the ELISA assay were performed and included the between-subjects factors TBI and PTZ. The statistical analysis revealed significant effects of TBI (*F*_(1, 28)_ = 253.0, *P* < 0.0001) and PTZ (*F*_(1, 28)_ = 30.19, *P* < 0.0001) for mPFC IL-1β; significant effects of TBI (*F*_(1, 28)_ = 84.12, *P* < 0.0001) and PTZ (*F*_(1, 28)_ = 9.021, *P* < 0.01) for IL-6; and significant effects of TBI (*F*_(1, 28)_ = 1003.0, *P* < 0.0001) and PTZ (*F*_(1, 28)_ = 11.47, *P* < 0.0001) and a significant effect of TBI × PTZ interaction (*F*_(1, 28)_ = 61.26, *P* < 0.0001) for TNF-α. Post-hoc analysis showed that IL-1β (*P* < 0.01, Fig. 5B), IL-6 (*P* < 0.05, Fig. 5C) and TNF-α levels (*P* < 0.01, Fig. 5D) in mice mPFC in the TBI group exposed to the PTZ injection were higher than those of the other groups. Moreover, we also measured the spleen weight, which is increased by inflammation starting, and found that both TBI (*P* < 0.001) and PTZ (*P* < 0.001) induced weight alterations of the spleen compared to the corresponding sham mice (Fig. 5E). However, no significant difference between saline and PTZ treatment in TBI mice was observed.

### Intra-mPFC oxytocin microinjection mitigates epilepsy induced by TBI

**Figure 6:**
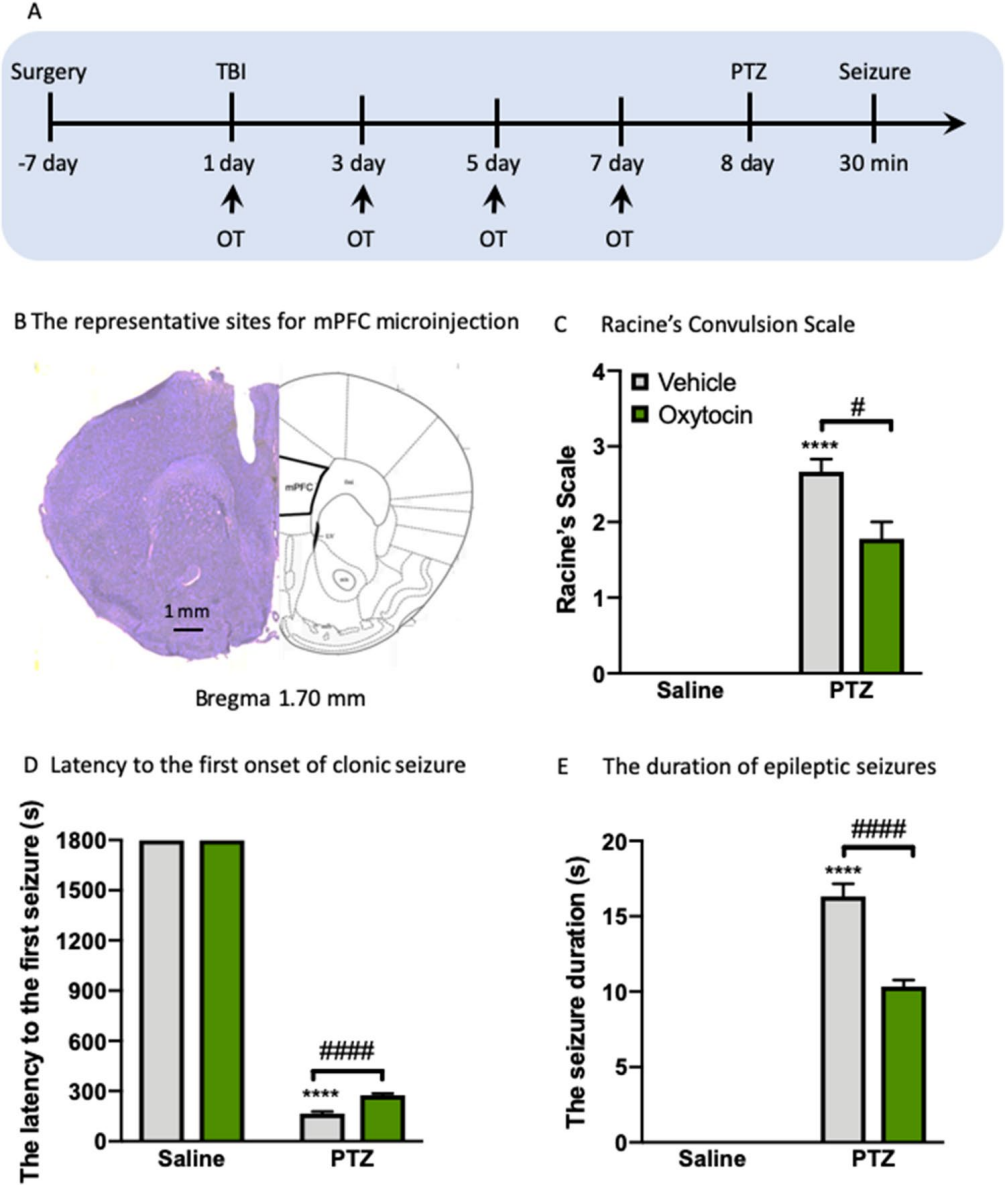
Intra-mPFC oxytocin microinjection mitigates epilepsy induced by TBI. (**A**) Experimental timeline of surgery and OT treatment. (**B**) Schematic coronal section showing the location used for oxytocin infusion into the mPFC. (**C**) The Racine’s Scale. (**D**) The latency to the first seizure. (**E**) The duration of epileptic seizures used for measurement of epilepsy in mice after TBI. Data are presented as mean ± SEM. ****P < 0.0001, compared with sham group; ^#^*P* < 0.05, ^####^*P* < 0.0001, compared with saline group. Two-way ANOVA followed by Turkey’s post hoc test. Non-parametric analysis followed by Kruskal–Wallis test was used for Racine’s Scales data. n = 8–9 per group. *TBI* traumatic brain injury, *PTZ* pentylenetetrazole, *NORT* novel object recognition test, *SRT* social recognition test, *OT* oxytocin.

Since the reduced levels of mPFC oxytocin is linked to the epilepsy performance, it is possible that enhancement of oxytocin in the mPFC will affect the subsequent outcome in TBI mice. The increase in neuroinflammation in the mPFC after TBI exposure to PTZ treatment may be mediated by the dysfunction of oxytocin system. To further investigate the potential role of oxytocin underlying the epilepsy in mice exposed to TBI, we assessed whether exogenous oxytocin supplementation could alleviate the development of epilepsy induced by TBI. For this purpose, we infused oxytocin or its vehicle into the mPFC after TBI and before the PTZ injection for 4 times within 8 days (Fig. 6A,B). mPFC infusions of oxytocin in PTZ-injected TBI mice showed that the epilepsy was reversed as reflected by reduced Racine’s Scale (*P* < 0.05, Fig. 6C), increased latency to the first seizure (*P* < 0.0001, Fig. 6D) and decreased seizure duration (*P* < 0.0001, Fig. 6E) by post-hoc analysis with Tukey correction for multiple comparisons. The two-way ANOVA revealed significant effects of PTZ (*F*_(1, 31)_ = 41,895, *P* < 0.0001) and oxytocin (*F*_(1, 31)_ = 50.51, *P* < 0.0001) and a significant effect of PTZ × oxytocin interaction (*F*_(1, 31)_ = 50.51, *P* < 0.0001) on the latency to the first seizure; and significant effects of PTZ (*F*_(1, 31)_ = 751.5, *P* < 0.0001) and oxytocin (*F*_(1, 31)_ = 38.05, *P* < 0.0001) and a significant effect of PTZ × oxytocin interaction (*F*_(1, 31)_ = 38.05, *P* < 0.0001) on the seizure duration.

### Intra-mPFC oxytocin microinjection mitigates cognitive impairments induced by TBI in PTZ-treated mice

**Figure 7:**
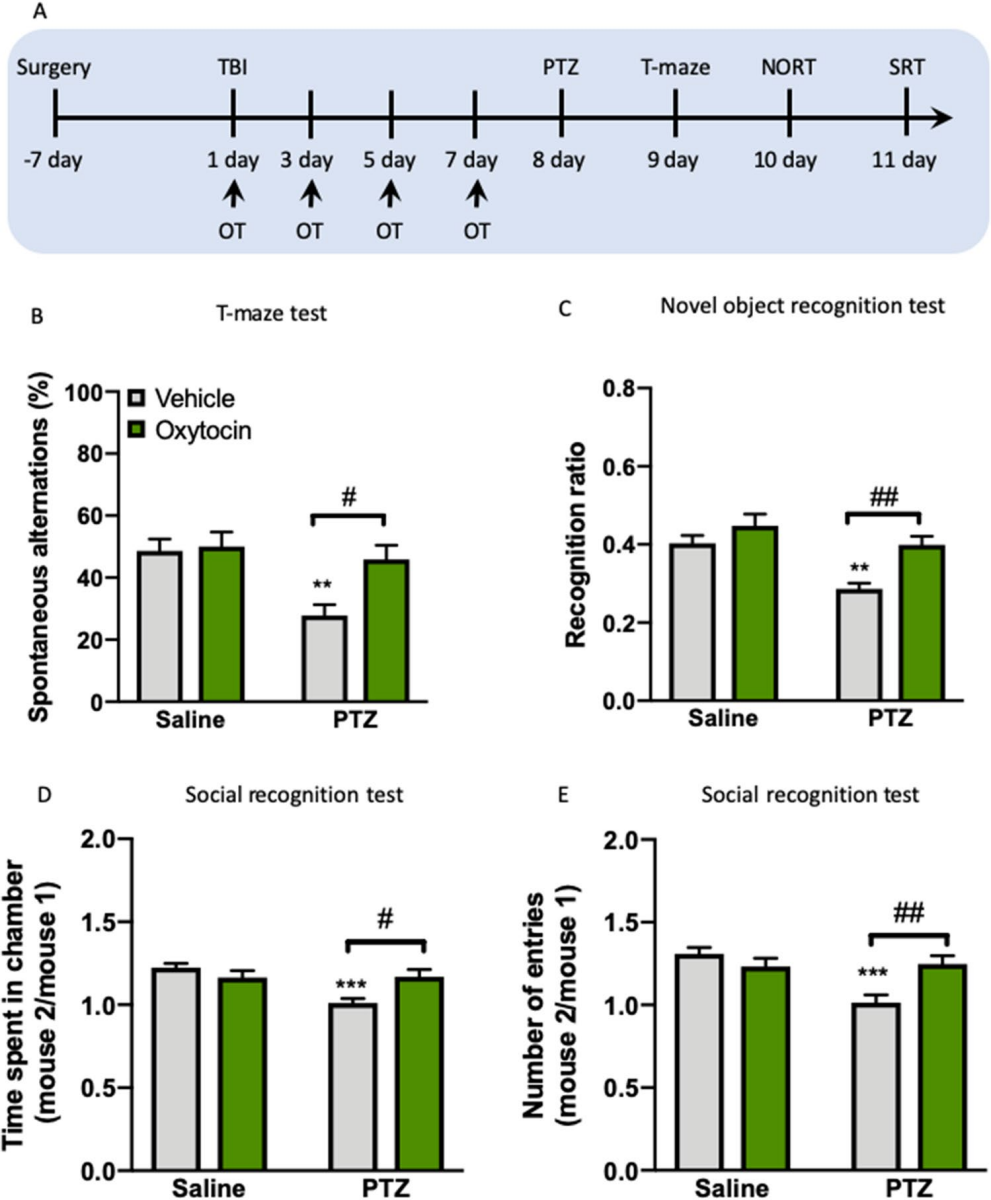
Intra-mPFC oxytocin microinjection mitigates cognitive impairments induced by TBI. (**A**) Experimental timeline of surgery and OT treatment. (**B**) The spontaneous alterations in T-maze test. (**C**) The recognition ratio in NORT. (**D**) Time spent in chamber in SRT. (**E**) The number of entries in SRT. Data are presented as mean ± SEM. **P < 0.01, ***P < 0.001, compared with sham group; ^#^*P* < 0.05, ^##^*P* < 0.01, compared with saline group. Two-way ANOVA followed by Turkey’s post hoc test. n = 8–9 per group. *TBI* traumatic brain injury, *PTZ* pentylenetetrazole, *NORT* novel object recognition test, *SRT* social recognition test, *OT* oxytocin.

We next detected the cognitive outcomes after infusion of oxytocin into the mPFC after TBI and before the PTZ injection to directly test the hypothesis that oxytocin could mitigate cognitive impairments (Fig. 7A). We found that mPFC infusions of oxytocin improved the cognitive performance as measured by increased spontaneous alterations in T-maze (*P* < 0.05, Fig. 7B) and recognition ratio in NORT (*P* < 0.05, Fig. 7C) in PTZ but not vehicle-treated mice. Data were analyzed using the factors PTZ (0 and 50 mg) and oxytocin (0 and 1 μg). This analysis revealed significant effects of PTZ (*F*_(1, 31)_ = 8.887, *P* < 0.01) and oxytocin (*F*_(1, 31)_ = 5.376, *P* < 0.05) and a close significant trend of PTZ × oxytocin interaction (*F*_(1, 31)_ = 3.950, *P* = 0.056) on the spontaneous alterations in T-maze. Two-way ANOVA revealed significant effects of PTZ (*F*_(1, 31)_ = 14.27, *P* < 0.001) and oxytocin (*F*_(1, 31)_ = 12.78, *P* < 0.01) on the recognition ratio in NORT. It is important to note that several studies have shown that oxytocin influences social recognition, which is an essential ability to differentiate individuals belonging to the same social group from unfamiliar individuals in mice^51,52^. Consistently, in the present study, we found that mPFC infusions of oxytocin increased the time spent (*P* < 0.05, Fig. 7D) and entry numbers (*P* < 0.05, Fig. 7E) in SRT, showing an improved social recognition. The statistical analysis revealed a significant effect of PTZ (*F*_(1, 31)_ = 8.973, *P* < 0.001) and a significant effect of PTZ × oxytocin interaction (*F*_(1, 31)_ = 9.797, *P* < 0.01) on the time spent in chamber and a significant effect of PTZ (*F*_(1, 31)_ = 9.028, *P* < 0.001) and a significant effect of PTZ × oxytocin interaction (*F*_(1, 31)_ = 10.98, *P* < 0.01) on the entry numbers in SRT. These data suggested that the epilepsy sensitivity and cognitive impairments in PTZ-treated TBI mice were both reversed by intra-mPFC oxytocin administration.

### Intra-mPFC oxytocin microinjection restores BBB integrity and reduces neuroinflammation

**Figure 8:**
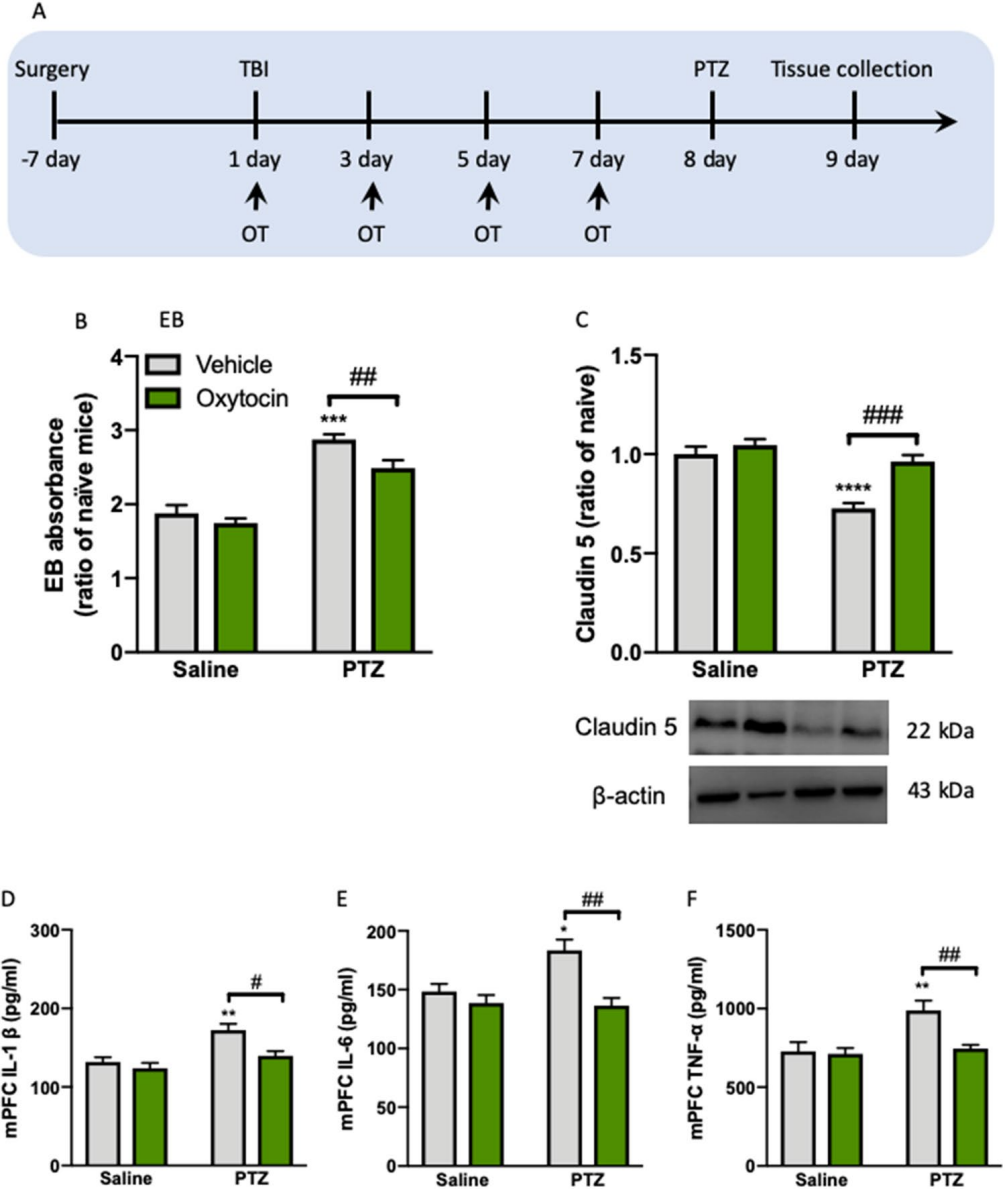
Intra-mPFC oxytocin microinjection restores BBB integrity and reduces neuroinflammation. (**A**) Experimental timeline of surgery and OT treatment. (**B**) The EB absorbance. n = 8 per group. (**C**) The Claudin-5 levels with representative images in Western blot on the bottom. n = 6 per group. The levels of (**D**) IL-1β, (**E**) IL-6 and (**F**) TNF-α in the plasma measured by ELISA Kit. n = 8 per group. Data are presented as mean ± SEM. **P < 0.01, ***P < 0.001, ****P < 0.0001, compared with sham group; ^#^*P* < 0.05, ^##^*P* < 0.01, ^####^*P* < 0.0001, compared with saline group. Two-way ANOVA followed by Turkey’s post hoc test. *TBI* traumatic brain injury, *PTZ* pentylenetetrazole, *OT* oxytocin.

The results linked the BBB integrity and neuroinflammation to seizure activity and cognitive deficits in TBI mice received PTZ. Therefore, we next tested whether the enhancement of oxytocin in the mPFC is necessary to restore BBB integrity and reduce neuroinflammation as well as contributes to the the beneficial effects of oxytocin on the epilepsy and cognition (Fig. 8A). mPFC infusions of oxytocin reduced EB absorbance in TBI mice exposed to PTZ treatment (*P* < 0.01, Fig. 8B). The statistical analysis revealed significant effects of PTZ (*F*_(1, 31)_ = 93.23, *P* < 0.0001) and oxytocin (*F*_(1, 31)_ = 8.304, *P* < 0.01) on the EB staining. Western blot assays showed that intra-mPFC infusions of oxytocin increased the Claudin-5 protein levels in PTZ-treated TBI mice compared to vehicle group (Fig. 8C). Data analysis revealed significant effects of PTZ (*F*_(1, 20)_ = 32.14, *P* < 0.0001) and oxytocin (*F*_(1, 20)_ = 20.19, *P* < 0.01) and a significant PTZ × oxytocin interaction (*F*_(1, 20)_ = 9.28, *P* < 0.01) on Claudin-5. The full-length uncropped Western blots are shown in Supplementary Information. Previous studies have shown that the inflammation is involved in the pathophysiology of both TBI and epilepsy, with increased levels of inflammatory cytokines. We attempted to provide direct evidence that intra-mPFC oxytocin regulates the inflammatory process that occurs in TBI-induced epilepsy and cognitive deficits. The blood samples from mice were collected for ELISA measurements of and IL-1β, IL-6 and TNF-α levels. We found that TBI treatment significantly increased IL-1β (*P* < 0.0001, Fig. 8D), IL-6 (*P* < 0.01, Fig. 8E), and TNF-α (*P* < 0.01, Fig. 8F) at the periphery, and intra-mPFC oxytocin blocked these activated cytokines of IL-1β (*P* < 0.01), IL-6 (*P* < 0.05), and TNF-α (*P* < 0.01) (Fig. 8D–F). The statistical analysis revealed significant effects of PTZ (*F*_(1, 28)_ = 16.99, *P* < 0.001) and oxytocin (*F*_(1, 28)_ = 9.016, *P* < 0.01) on IL-1β; significant effects of PTZ (*F*_(1, 28)_ = 5.021, *P* < 0.05) and oxytocin (*F*_(1, 28)_ = 15.08, *P* < 0.001) and a significant PTZ × oxytocin interaction (*F*_(1, 28)_ = 6.401, *P* < 0.05) on IL-6; and significant effects of PTZ (*F*_(1, 28)_ = 9.222, *P* < 0.01) and oxytocin (*F*_(1, 28)_ = 7.182, *P* < 0.01) and a significant PTZ × oxytocin interaction (*F*_(1, 28)_ = 5.398, *P* < 0.05) on TNF-α. Together, these findings indicate that, among the beneficial functions of oxytocin in improving epilepsy and cognition, BBB integrity and neuroinflammation in the mPFC play a significant role in the reversal of TBI-triggering negative behaviours.

## Discussion

In the present study, we found that TBI exposure increases the vulnerability to PTZ-mediated seizure activity and causes cognitive impairments in mice. The breakdown of BBB integrity increases the passage of peripheral inflammatory cytokines to the mPFC and is associated with seizure severity and cognitive decline. In addition, the BBB disruption was accompanied by loss of tight junction protein Claudin-5 and subsequently lead to neuroinflammation in the mPFC. Oxytocin infused into the mPFC after TBI is sufficient to suppress the seizure vulnerability and to rectify the cognitive deficits in mice. Simultaneously, PTZ-altered pattern of Claudin-5 expression and BBB permeability was blocked by treatment with oxytocin in the mPFC of TBI mice. Furthermore, the reduced transfer of inflammatory cytokines IL-1β, IL-6 and TNF-α from the circulation to the brain was also involved in the reversal effects of oxytocin on the epileptic sensitivity and impaired cognition in mice previously experienced TBI. We confirmed that BBB integrity and neuroinflammation blockage in the mPFC mediated the behavioral improvement of oxytocin by reducing inflammatory cytokines reaching the central nervous system. Altogether, our findings may provide insight into the clinical translation of neuropeptide oxytocin that is promisingly implicated in treating epilepsy and cognitive decline occurring after traumatic brain injury via BBB enhancement in the medial prefrontal cortex.

The mortality of patients suffered epilepsy after traumatic brain injury is dramatically increased as much as three times compared with those not developing epilepsy^53^. Moreover, cognitive deficit is one of the long-term consequences of TBI affecting individuals’ health and quality of life and increases risk of developing Alzheimer's disease in later life^54^. Although multiple health concerns associated with TBI have been widely characterized by accumulating evidence, there are no available animal models that fully recapitulate the incidence of seizure sensitivity and cognitive impairment after TBI. Therefore, establishment of effective animal models that are specifically repetitive to behavioral and neurological consequences related to brain injury can help to find translational managements and interventions for these poor outcomes. In the present investigation, we used mouse weight-drop model that has also been shown to replicate human TBI symptoms. The specie of C57/BL6J mice we used for the current research was based on previous evidence showing that these mice are reliable and sensitive to drug-induced epilepsy with abnormal neuronal damage and inflammation in the brain, and they also demonstrated translational phenotypes in cognitive performance. To mimic epilepsy associated with TBI, we injected mice with PTZ 7 days following weight-drop injury to trigger seizure. Furthermore, we demonstrate through multiple indicators including Racine’s Scale, latency to the first onset of clonic seizure, and the duration of epileptic seizures in this model that oxytocin microinjected into the mPFC can decrease the seizure activity after TBI. In order to ascertain the possibility that the loss of cognition could be improved by oxytocin, we selected different tasks to map diverse aspects of cognitive ability, for example, working memory is tested primarily using T-maze, episodic memory using novel object recognition task, and social learning process is defined using social recognition test according to previous descriptions^55,56^. From these series of tasks, we observed that TBI mice received PTZ demonstrated exacerbated cognitive performance while intra-mPFC administration of oxytocin for 7 days after TBI restored these negative outcomes in cognition.

It is well established that seizure and epilepsy correlate with BBB dysfunction and breakdown in experimental animal models^49^. Adding this, the weight-drop TBI model we used here can also cause BBB disruptions as shown by EB absorbance value. In the present study, we aimed to investigate whether the beneficial effects of oxytocin on the seizure and cognition are due to the re-stabilization of BBB integrity which is disrupted by TBI. Claudin-5, one of tight junction proteins, is the critical component expressed in brain endothelial cells, and loss of Claudin-5 in key brain regions has been shown to decrease BBB tightness and increase permeability^57,58^. For example, inducible Claudin-5 knockdown mice develop spontaneous seizures via activation of endothelial cells^49^. Moreover, downregulation of Claudin-5 expression in the nucleus accumbens is associated with depression-like phenotypes^59^. Similarly, we found a significant relationship between BBB integrity and epilepsy severity or cognitive decline. Meanwhile, Claudin-5 protein expression in the mPFC has been demonstrated to be decreased, suggesting that enhancement of BBB stability might be helpful to normalize seizure activity and cognitive function in TBI mice. Interestingly, intra-mPFC administration of oxytocin induce a significant increase of Claudin-5 protein levels accompanied with a reduction of EB staining that can reduce epilepsy development and improve cognitive impairments.

Cumulative evidence suggests that neuroinflammation featured as release of endogenous danger signals and innate immune activation may be a major contributor in the etiology and recovery of TBI and consequence of post-traumatic epilepsy^8,60^. In addition, the blocking pro-inflammatory factors as well as enhancing oxytocin function play a key role in the pathogenesis and therapy of TBI^61^. An improved understanding of how inflammation contributes to epileptogenesis after TBI is key to the development of cytokine-targeted therapies, aiming to alleviate secondary damage as well as prevent epilepsy development after TBI. We find here that TBI significantly increased pro-inflammatory IL-1β, IL-6 and TNF-α at the periphery, while intra-mPFC oxytocin blocked these activated cytokines. Since the neuroinflammatory response to TBI appears to be largely pro-epileptogenic, further research is needed to clearly causal relationship between oxytocin and neuroinflammation. Notably, the role of TNF-α signaling in epilepsy that develops after TBI has not yet been identified. The present understanding is that TNF-α demonstrates conflicting roles in both anti-epileptic and pro-epileptic activity, depending on binding different receptors. It has been evidenced that the anti-epileptic activity of TNF-α is mediated by the p75 pathway, while the p55 pathway is involved in the pro-epileptic activity of TNF-α^62^. In the context of epilepsy, elevated levels of IL-6 in both brain and plasma have been implicated in the severity of seizures in both patients with epilepsy conditions and animal studies^63,64^. We propose that the increase of neuroinflammation in the mPFC is resulting from BBB disruption, which has been evidenced to promote the passage of blood circulating pro-inflammatory cytokines that can then penetrate the brain to participate in the development of epilepsy.

The finding that TBI mice are more prone to seizure activity and cognitive decline illustrates the importance of investigating brain activity or functional changes involved in this process so as to provide clues for preventing this disorder. It has shown that oxytocin neurons are expressed in the paraventricular (PVN) of the hypothalamus and also project to several brain regions playing key roles in regulating memory and social behaviour, such as frontal cortex and amygdala^23,65,66^. Additionally, previous evidence has displayed an overall decrease in brain oxytocin levels in a mouse model of focal epilepsy syndrome^67^, demonstrating a potential role for dysregulation of the oxytocin system in behavioral deficits related to epilepsy. Intriguingly, we show that pharmacological manipulation of the oxytocin system after traumatic brain injury upregulates oxytocin function and changes epilepsy and cognitive outcomes in PTZ-evoked mice. Several studies evidenced the impairment and dysfunction of deep layer neurons in the mPFC is responsible for the seizure onset and associated cognitive decline^68,69^. Moreover, psychoactive compounds used for the treatment of epilepsy modulates the activity of excitatory amino acid and neuronal spontaneous activity of a specific population of pyramidal neurons in the mPFC^70^. An increasing body of research points toward the spontaneous activity of the mPFC pyramidal neurons are modulated mainly by interactive excitation between glutamatergic pyramidal neurons and regulated by inhibitory GABAergic interneurons to maintain the mPFC^71,72^. It is worth to note that oxytocin attenuated the levels of glutamate and stimulated inhibitory neurotransmitter GABA in the mPFC induced by psychostimulants^73^, suggesting that mPFC oxytocin may serve as a pharmacotherapeutic candidate in the treatment of post-traumatic epilepsy and cognitive dysfunction. In the present study, oxytocin infused into the mPFC significantly mitigates epilepsy and cognitive impairments induced by TBI. These findings suggest that the restoration of mPFC oxytocin activity might be a potential neural substrate contributed to the reversal of epileptic development and cognitive dysfunction. Given that oxytocin receptors (OTR) are expressed in the mPFC^74,75^ and receptors activation is required for oxytocin to regulate neurobiological functions, it is possible that activation of the OTR might also be essential for mPFC oxytocin to mitigate epilepsy and cognitive impairments in PTZ-triggered TBI mice. Fox example, oxytocin altered the reward memory of psychostimulant methamphetamine via OTR in mPFC in mice^76^, as well as oxytocin reduced anxiety-like behavior when delivered to the mPFC by activating the OTR in rats^77^. However, our results did not measure the changes of OTR levels and did not determine the effects of OTR antagonist after oxytocin treatment, so we could not provide direct experimental evidence supporting the potential role of OTR in cognitive process after exogenous mPFC oxytocin injection. More specific strategy aiming to clarify the regulatory role of oxytocin/OTR signalling should be explored in the future.

Seizures occurring after traumatic injury are caused by excessive neuronal activity due to the disruption of excitation-inhibition balance in the brain^78^. A loss of inhibitory GABAergic interneurons output in mice may underlie the generation and maintenance of seizures^79^. In addition to inhibitory neurotransmitter, the excess release of glutamate, a predominant excitatory neurotransmitter, also contributes to the aberrant neuronal activity under conditions of recurrent seizures and chronic epilepsy. Here, we established mice epilepsy and cognitive decline model by PTZ injection after TBI. Previous evidence has identified that PTZ leads to a decrease in GABAergic function and a stimulation of glutamate receptor subtypes, and consequently results in an increased activity of glutamatergic systems in PTZ-induced seizure models^24,80^. Notably, oxytocin suppresses glutamatergic neurotransmission in the infralimbic medial prefrontal cortex layer pyramidal neurons by decreasing the release of glutamates^29^. Therefore, the decreased levels of oxytocin in the mPFC that we presented here is possible due to the failure in suppressing glutamatergic neurotransmission, which is stimulated by PTZ-induced seizure. It is important to note that intra-mPFC oxytocin administration reversed the seizure vulnerability, confirming our hypothesis that supplementation of exogeneous oxytocin is sufficient to inhibit seizures via normalization of the glutamatergic activation especially in the mPFC. Although we did not directly measure the glutamate in the present study, the possible mechanism underlying the anti-seizure effects of oxytocin has been evidenced by suppressing glutamatergic neurotransmission in the medial prefrontal cortex^29^. Nevertheless, the measurement of glutamate is still needed in the future to characterize the relationship between behavioral improvements and glutamate release after oxytocin treatment. Another point that deserves further attention is whether endogenous oxytocin activation is also effective to rescue the epileptic patterns and cognitive deficits. Regarding this, additional studies are underway to cause endogenous oxytocin release, such as by a selective melanocortin receptor 4 agonist, and to evaluate the influences of endogenous oxytocin on epilepsy and cognition altered by TBI. This identification will narrow the knowledge gaps in health conditions resulted from TBI and improve understanding and awareness of the positive consequences of oxytocin.

**Figure 9:**
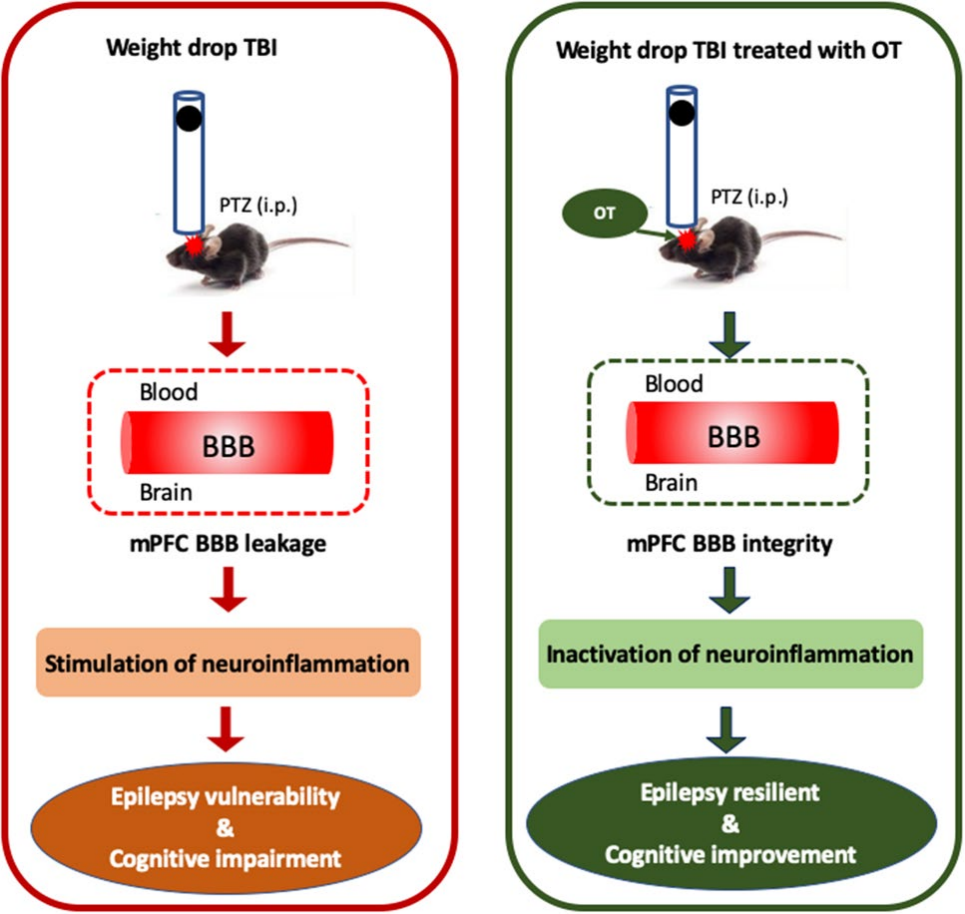
The outline representing mPFC oxytocin mitigates epilepsy and cognitive impairments induced by TBI. Previous TBI exposure disrupts BBB integrity and subsequently stimulates neuroinflammation in the mPFC, leading to the increased vulnerability to epilepsy and cognitive dysfunction. Intra-mPFC oxytocin administration inhibits BBB leakage and inactivates neuroinflammation (shown by reduced inflammatory cytokines IL-1β, IL-6 and TNF-α) and eventually reverses epileptic development and cognitive dysfcunction. *TBI* traumatic brain injury, *PTZ* pentylenetetrazole, *mPFC* medial prefrontal cortex, *BBB* blood–brain barrier.

In conclusion, we demonstrate that oxytocin suppresses the seizure vulnerability as well as rescues the cognitive deficits when delivered directly into the mPFC of TBI mice that exposed to PTZ. The normalization of BBB integrity and subsequent neuroinflammation inhibiting may be involved in the antiepileptic and cognition-improved effects of oxytocin. The results of the current study add to growing evidence that enhancement of BBB properties is beneficial to treat epilepsy and other neurological conditions including cognitive dysfunctions. Future work targeting BBB and related components in specific brain region may reduce the risk to develop epilepsy and cognitive impairments in individuals previously experienced TBI (Fig. 9).

## Supplementary Information

Supplementary Information 1.

Supplementary Information 2.

Supplementary Information 3.

Supplementary Information 4.

Supplementary Information 5.

Supplementary Information 6.

## Data Availability

The datasets used and/or analysed during the current study available from the corresponding author on reasonable request.
