# Supplementary figures and images for: Medial prefrontal cortex oxytocin mitigates epilepsy and cognitive impairments induced by traumatic brain injury through reducing neuroinflammation in mice
Source: Sci Rep. 2023 Mar 30;13:5214. doi: 10.1038/s41598-023-32351-8 (PMC10063625; doi:10.1038/s41598-023-32351-8)

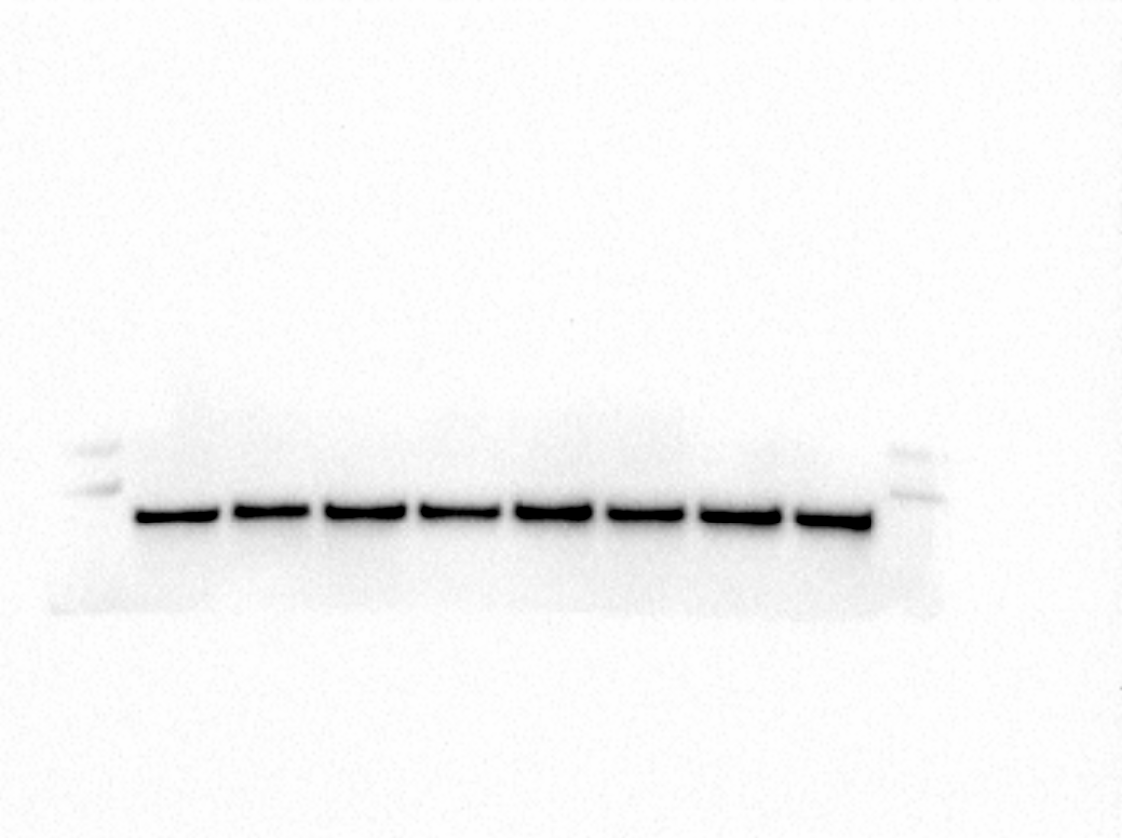

Supplement: Supplementary file 1 — Supplementary Information 1. [file 41598_2023_32351_MOESM1_ESM.tif]

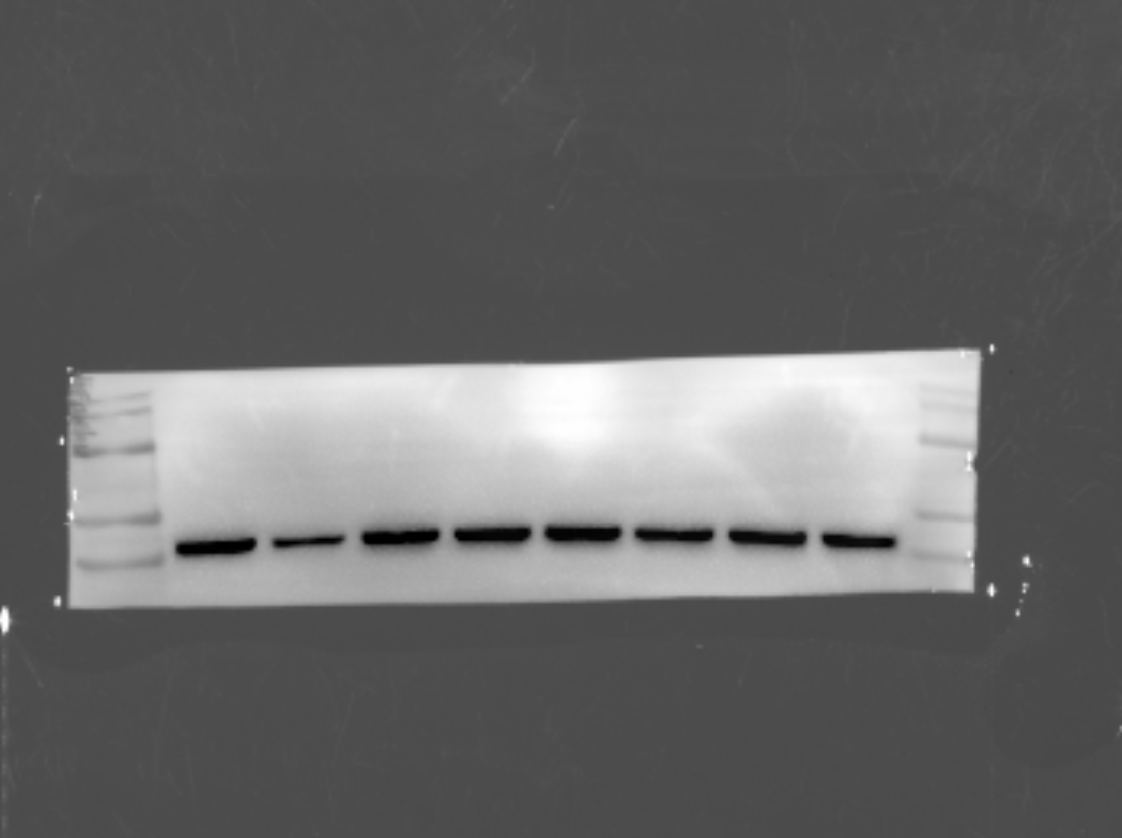

Supplement: Supplementary file 2 — Supplementary Information 2. [file 41598_2023_32351_MOESM2_ESM.tif]

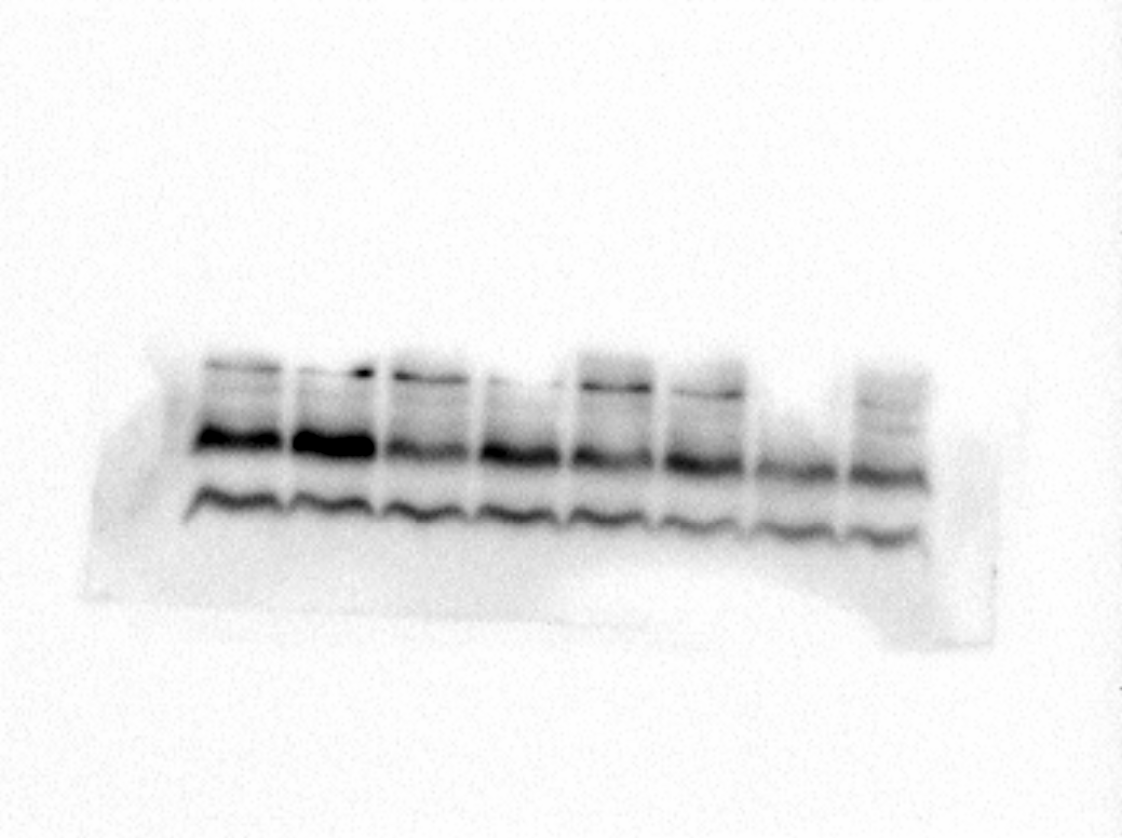

Supplement: Supplementary file 3 — Supplementary Information 3. [file 41598_2023_32351_MOESM3_ESM.tif]

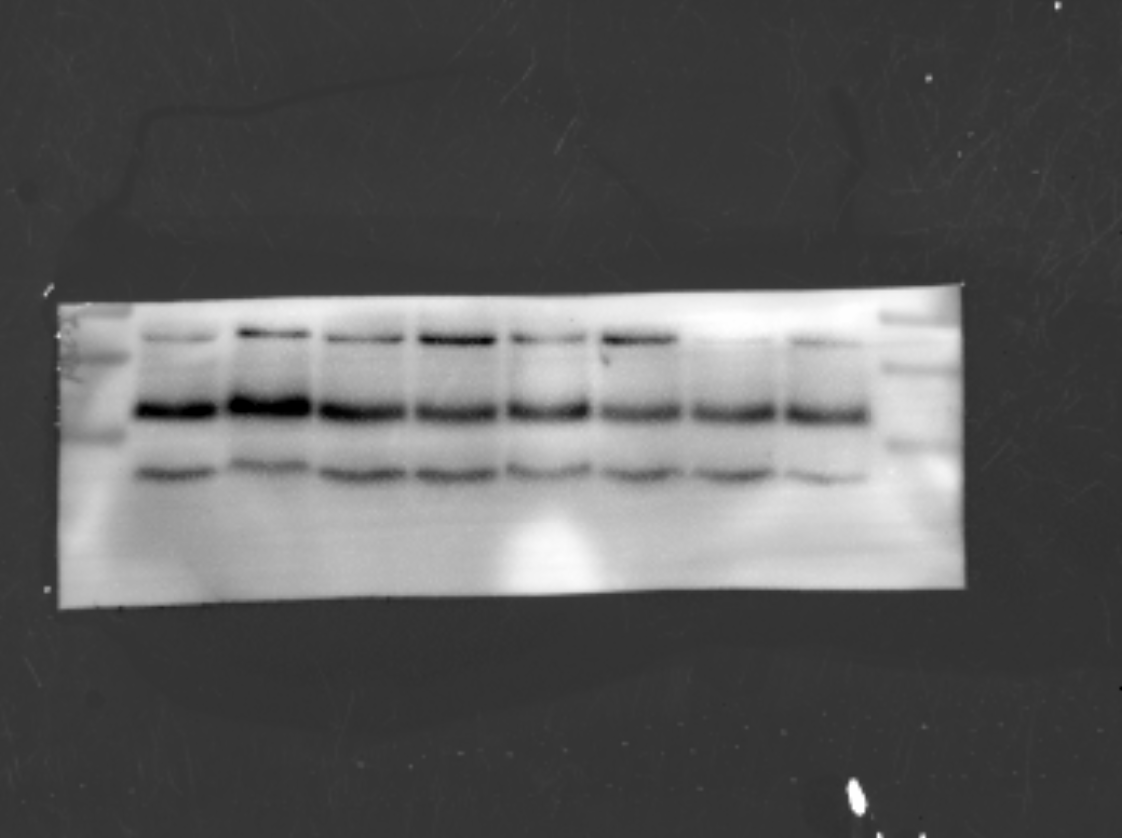

Supplement: Supplementary file 4 — Supplementary Information 4. [file 41598_2023_32351_MOESM4_ESM.tif]

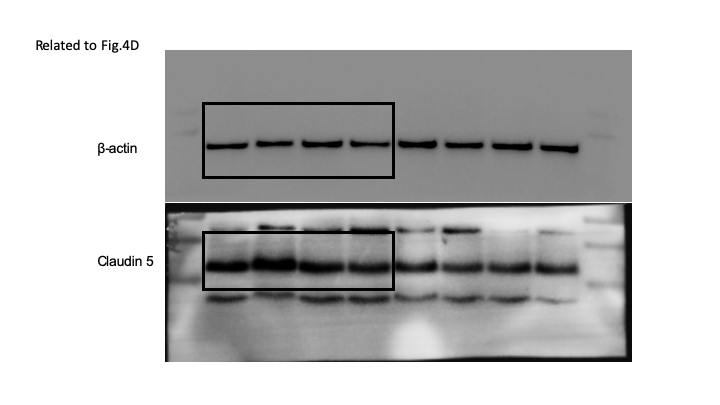

Supplement: Supplementary file 5 — Supplementary Information 5. [file 41598_2023_32351_MOESM5_ESM.tiff]

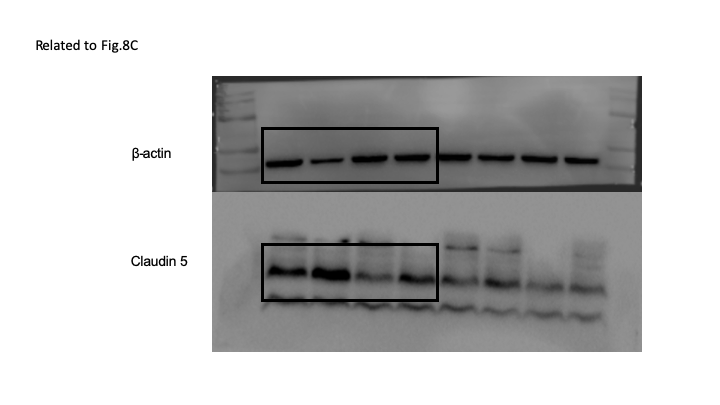

Supplement: Supplementary file 6 — Supplementary Information 6. [file 41598_2023_32351_MOESM6_ESM.tiff]
